# Recent progress in the synthesis of nanostructured Ti_3_C_2_T_*x*_ MXene for energy storage and wastewater treatment: a review

**DOI:** 10.1039/d5na00021a

**Published:** 2025-04-24

**Authors:** Qui Thanh Hoai Ta, Jianbin Mao, Ngo Thi Chau, Ngoc Hoi Nguyen, Dieu Linh Tran, Thi My Huyen Nguyen, Manh Hoang Tran, Hoang Van Quy, Soonmin Seo, Dai Hai Nguyen

**Affiliations:** a Institute of Advanced Technology, Vietnam Academy of Science and Technology 1A TL29 Street, Thanh Loc Ward, District 12 Ho Chi Minh City 700000 Vietnam nguyendaihai0511@gmail.com tathanhhoaiqui2292@gmail.com chaungo2601@gmail.com hoi83bmt@gmail.com tdlinh92@gmail.com myhuyen1001vn@gmail.com tranmanhhoang1214@gmail.com; b Graduate University of Science and Technology, Vietnam Academy of Science and Technology 18 Hoang Quoc Viet Street, Cau Giay District Hanoi 100000 Vietnam; c College of BioNano Technology, Gachon University Gyeonggi 13120 Republic of Korea soonmseo@gachon.ac.kr mg2895852@gmail.com; d Faculty of Pharmacy, Nguyen Tat Thanh University 300A Nguyen Tat Thanh Street, Ward 13, District 4 Ho Chi Minh City 700000 Vietnam; e Division of Energy & Environmental Technology, Daegu–Gyeongbuk Institute of Science and Technology (DGIST) Daegu 42988 Republic of Korea quybk@dgist.ac.kr

## Abstract

MXene-based functional 2D materials hold significant potential for addressing global challenges related to energy and water crises. Since their discovery in 2011, Ti_3_C_2_T_*x*_ MXenes have demonstrated promising applications due to their unique physicochemical properties and distinctive morphology. Recent advancements have explored innovative strategies to enhance Ti_3_C_2_T_*x*_ into multifunctional materials, enabling applications in gas sensing, electromagnetic interference shielding, supercapacitors, batteries, water purification, and membrane technologies. Unlike previous reviews that primarily focused on the synthesis, properties, and individual applications of MXenes, this work provides a fundamental discussion of their role in wastewater treatment, recent advancements in energy harvesting, and their broader implications. Additionally, this review offers a comparative analysis of MXene-based systems with other state-of-the-art materials, providing new insights into their future development and potential applications.

## Abstract 2

MXene-based functional 2D materials hold significant potential for addressing global challenges related to energy and water crises.

## Graphical abstract

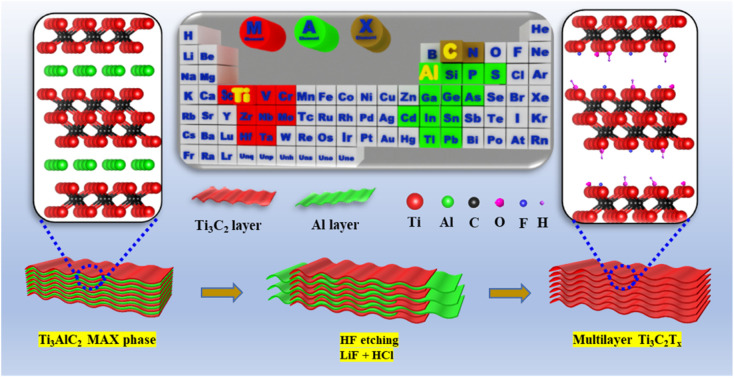

## 1. Introduction

An increasing population and industry have brought massive global changes in the form of water crises and energy scarcity. Water is typically utilized in energy-production procedures, while energy is needed for the purification of water resources and water remediation; thus, water issues and energy dilemmas are relevant and interdependent.^1–5^ Over 1.3 billion civilians are living without access to electrical power, and around 1.6 million civilians lack clean water for daily activities.^6,7^ These issues will be further worsened due to global warming and the consistent demand for clean water and energy, which has been predicted to increase by over 50% over the next decade.^8,9^ Additionally, untreated wastewater is threatening human health and natural ecosystems. Moreover, current energy storage and conversion technologies are not suitable because of the inefficiency of these systems.^10,11^

Scientific communities have studied materials and technology to solve the aforementioned issues, among which two-dimensional (2D) materials have attracted tremendous research attention owing to their unique properties and tunable structures. In particular, the discovery of carbides and nitrides (MXenes) in the 21st century further boosted the intensity of breakthrough research associated with carbon-based functional materials.^12–15^ MXenes are a group of 2D materials that have been explored since Gogotsi and co-workers reported them in 2011.^14^ Ti_3_C_2_T_*x*_ MXenes have synthesized by the etching of Al-containing MAX phases, where M is a pre-transition metal (Mo, Ta, Hf, Cr, Ti, V, *etc.*), A represents an A-group element (groups 13 and 14, or IIIA and IVA), and X stands for either C and/or N.^16^ The etched A element is usually replaced by a termination group (–F, –O, and –OH), giving MXene materials with a common structure such as M_*n*+1_X_*n*_T_*x*_ with *n* = 1 − 4.^17,18^

Ti_3_C_2_T_*x*_ MXenes are crucial materials due to their intrinsic properties, including high electrical conductivity, hydrophilicity, and excellent mechanical strength. Consequently, they have been widely utilized in various applications, such as electrodes, energy storage materials, and co-catalysts in photocatalysis. Ti_3_C_2_T_*x*_ films can be fabricated using multiple techniques, including vacuum-assisted filtration, spin-coating, rolling, printing, and spray-coating of exfoliated MXene solutions.^19^ Various properties and potential applications of Ti_3_C_2_T_*x*_ MXenes have been investigated in the literature.^20–28^ However, there has been limited research into the reuse and recycling of Ti_3_C_2_T_*x*_ materials from used supercapacitors and spent batteries. It is important to investigate the recycling of spent Ti_3_C_2_T_*x*_ MXene, as this would facilitate a broad range of applications and increase their environmental friendliness. Innovative methods for MXene-based multi-functional materials are expected to improve the synthesis cost of materials for energy generation and mitigate the global warming caused by wastewater. Driven by investigations into methods, efficiency, and stability, the synthesis of valuable MXene-based multi-functional materials is anticipated to bring about a sustainable future.

Numerous reviews have explored the diverse applications of MXenes, including their roles in electromagnetic interference shielding, gas sensing, photocatalysis, electrochemical energy storage systems, regenerative medicine, and next-generation rechargeable batteries.^29–35^ While earlier reviews have primarily emphasized the synthesis and singular applications of MXenes, the present work focuses on their integrated applications in energy and environmental domains. It highlights recent advancements, advanced characterization techniques, and scalability challenges associated with Ti_3_C_2_T_*x*_ MXenes. Furthermore, this review provides a fundamental discussion of electron transfer mechanisms, offers a critical comparison between MXene-based materials and other leading alternatives, and outlines key challenges and future directions for their practical deployment. The integration of these domains aligns with the global transition toward sustainable technologies. By examining the structure–function relationships of Ti_3_C_2_T_*x*_ MXene, this review seeks to demonstrate their multifunctionality and versatility, ultimately supporting the development of advanced platforms to address critical issues related to the global energy and water crises.

## 2. Synthesis and properties of MXene

### 2.1. Synthesis

Since their initial exploration, more than 160 MAX phases have been studied using density functional theory (DFT) calculations and experimental works. Two major dilemmas are encountered in this exploration: the identification of appropriate precursors and the development of feasible synthetic methods at the scale-up stage.^36,37^

**Fig. 1:**
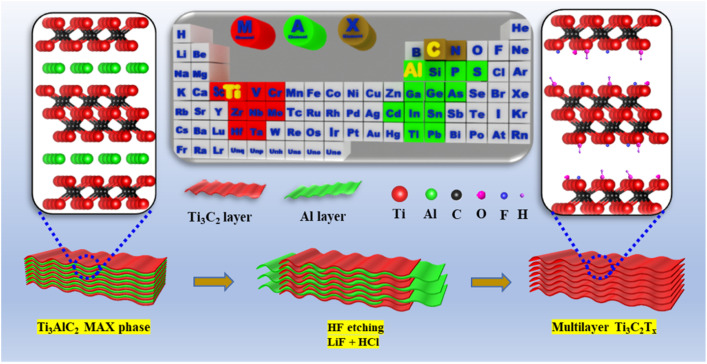
Schematic preparation processes of Ti_3_C_2_T_*x*_ MXene using a F-based method. Reproduced from ref. 39 with permission from Elsevier, copyright 2021.

There are two major methods to prepare Ti_3_C_2_T_*x*_ MXenes: bottom-up and top-down methods. The top-down method is usually used to prepare MXene owing to its ease of scale-up, simple equipment, and cheapness compared to the bottom-up technique. Ti_3_C_2_T_*x*_ MXenes have been synthesized using HF acid as an etching agent to remove the Al layer (Fig. 1).^38,39^

However, scientists have become concerned about the toxicity of HF, and efforts have been made to find other milder etching procedures instead of using HF, such as electrochemical etching, alkaline etching using NaOH, HCl and LiF, and molten salt.^40–44^ The methods allow for control of the nano- and micro-size of MXene, but require complicated systems with a small number of products. Therefore, the manufacture of Ti_3_C_2_T_*x*_ MXene has been dominated by etchants at the laboratory and factory scale. In particular, the use of hazardous HF acid to produce MXene seems to be difficult for mass production. To date, there are no viable alternatives to HF and *in situ*-formed HF as etchants; this challenge is in the embryonic stage. It is possible to synthesize the desired Ti_3_C_2_T_*x*_ MXene with proper granulometry, colloidal systems, and free-standing films, which are suitable for specific applications.^45–47^

**Fig. 2:**
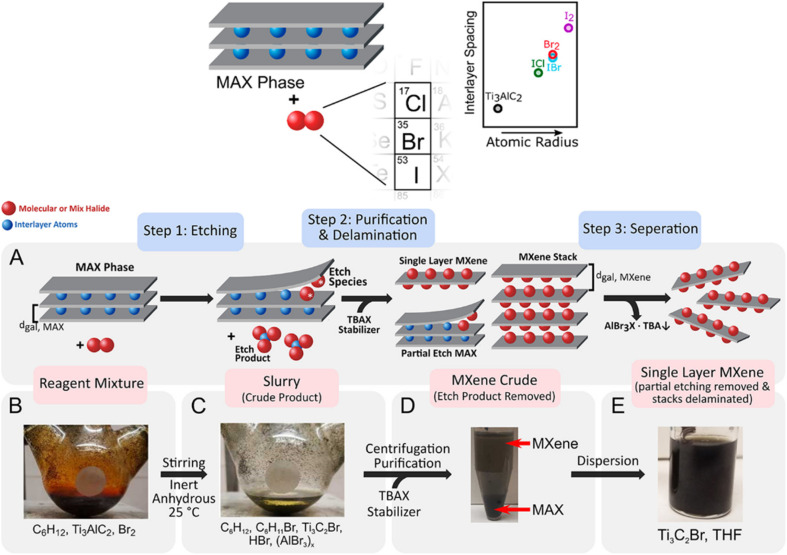
(A–E) Halogen etching of Ti_3_AlC_2_ MAX phase. Reproduced from ref. 48 with permission from American Chemical Society, copyright 2021.

Halogen-based etching has recently been used in the synthesis of Ti_3_C_2_T_*x*_ MXenes with halogen-terminated surfaces, as shown in Fig. 2. The rate and extent of removal can be controlled either optically or qualitatively owing to its colorimetric parameters, which offer direct quantitative feedback as compared to fluoride-based techniques. The plausible mechanism reveals that continual halogen (I_2_, Br_2_) injection offers high yields and efficiency (∼1% yield Ti_3_C_2_T_*x*_ at 1 mg mL^−1^).^48^

**Fig. 3:**
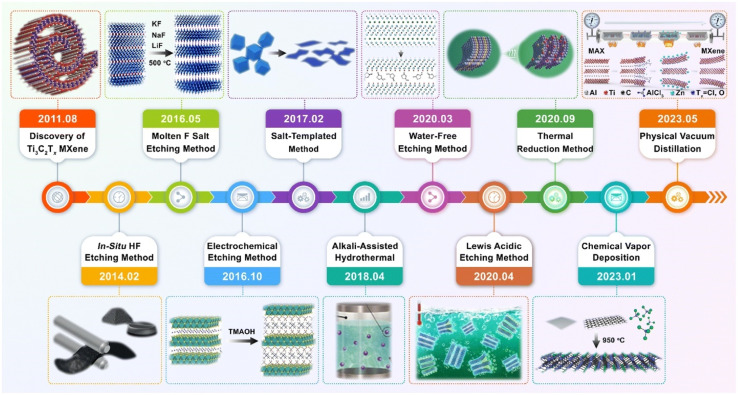
Schematic of synthesis Ti_3_C_2_T_*x*_ MXene with variety of techniques. Reproduced from ref. 49 with permission from the Royal Society of Chemistry, copyright 2023.

*In lieu* of HF, science communities have modified the etchant to obtain MXenes with variety of physicochemical properties that are suitable for practical applications. Fig. 3 presents a timeline of the preparation routes of MXene since its discovery.

### 2.2. Properties

The unique properties of Ti_3_C_2_T_*x*_ MXenes typically depend on their composition, lateral size, etchant, and stacking order. When the Al layers are removed from the Al-containing MAX phase, Ti layers are exposed on two sides of the MXene layer, which are prone to bond with functional groups (=O, –F, and –OH) to decrease the total surface energy. Thus, it is important to fundamentally understand the effect of functional groups on the unique properties of Ti_3_C_2_T_*x*_ so as to achieve versatility in designing applications.^50,51^

Unlike graphene, Ti_3_C_2_T_*x*_ MXenes have unique properties, such as high electronic conductivity, abundant terminal groups, and lamellar 2D structures. Electrical measurements were conducted on flakes and foam structures of metallic Ti_3_C_2_T_*x*_ MXenes, and gave values of around 10 000 S cm^−1^.^52^ The functional groups (F, OH, O) have an intrinsic oxidizing nature, which accelerates the redox reaction. Finally, the multilayered morphology allows high specific surface area and favors diffusion toward the active sites.^47,53^

#### 2.2.1 Thermal and chemical stability

The stability of metallic MXenes is crucial for potential applications, since samples may undergo a series of heat treatments during the preparation of devices and under specific working environments. In particular, thermal treatment in an Ar/N_2_ atmosphere at high temperature results in the elimination of functional groups and enhances the crystallinity without any defects in the multilayered morphology.^54^ Moreover, Ti_3_C_2_T_*x*_ is easily oxidized in an aqueous environment or hot air, transforming it into MXene-derived oxides.^55^ For example, Naguib *et al.* synthesized TiO_2_/carbon sheets simply *via* heat treatments of Ti_3_C_2_T_*x*_ MXene in air at 1150 °C for 30 s.^56^ This transformation results in some unique properties, typically decreased conductivity and superior pseudo-capacitance. Therefore, Ti_3_C_2_T_*x*_-based composites should be designed rationally depending on the potential applications.^57^

#### 2.2.2 Mechanical properties

**Fig. 4:**
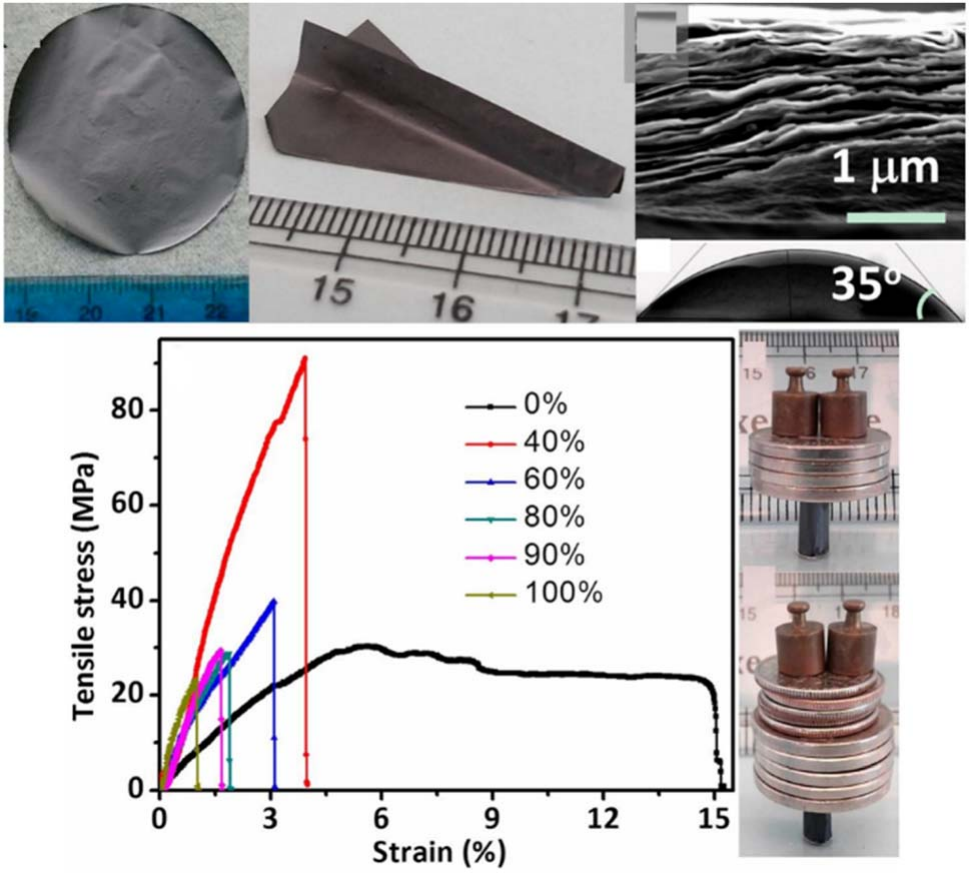
Mechanical properties of a metallic Ti_3_C_2_T_*x*_ MXene film. Reproduced from ref. 61 with permission from the National Academy of Sciences of the United States of America, copyright 2014.

The elasticity (*E*^2D^) was estimated to range from 278 to 393 N m^−1^ for single-layered MXenes based on Poisson's ratio, and from 632 to 683 N m^−1^ for two-layered Ti_3_C_2_T_*x*_ MXene.^58^ The average elasticity of mono-layered MXene (326 N m^−1^) was half that of two-layered MXene (655 N m^−1^), which was ascribed to the excellent interlayer interaction between functional groups. According to the nanoindentation method, Ti_3_C_2_T_*x*_ MXene exhibited a lower *E*^2D^ than h-BN and graphene, but higher than that of molybdenum sulfide (MoS_2_), reduced graphene oxide (rGO), and GO.^59^ Moreover, the Ti_3_C_2_T_*x*_ MXene was able to be rolled into a conical shape (*r* < 20 nm), which demonstrated its good flexibility.^60^ The Ti_3_C_2_T_*x*_ MXene-derived free-standing paper could be folded into an airplane model without any damage. A paper cylinder assembled from Ti_3_C_2_T_*x*_ MXene can support around 4000 times its own weight (Fig. 4).^61^ The combination of PVA and MXene further improved the mechanical strength of PVA@MXene composite by 33.5%, allowing it to bear around 15 000 times its own weight.^50^ The hierarchical architecture of the MXene-bonded polyurethane/polyvinyl alcohol (PU/PVA) hydrogel has the potential to enhance its both strain and mechanical strength characteristics; the material exhibited a gauge factor of 5.7 at a strain of 191% after undergoing 5000 cycles.^62^

#### 2.2.3 Electrocatalytic properties

**Fig. 5:**
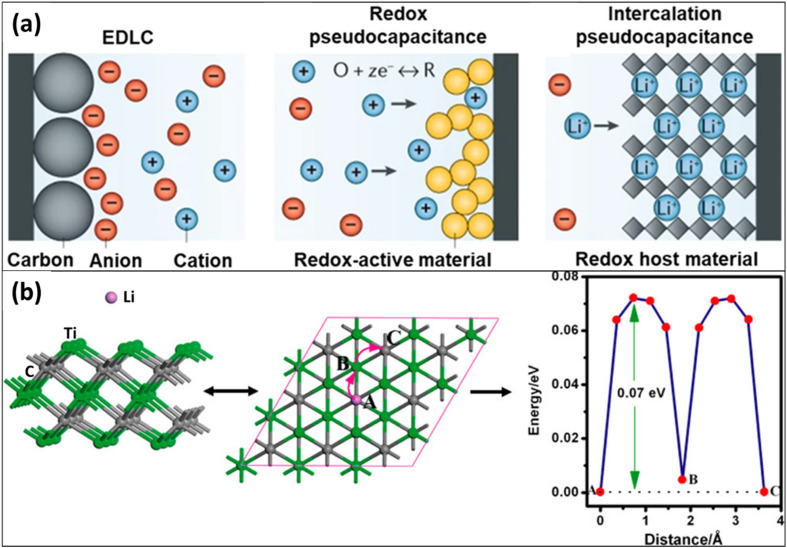
Schematic illustrations depicting (a) the mechanisms of electrochemical charge storage and (b) the pathways for lithium diffusion within Ti_3_C_2_T_*x*_ MXene. Reproduced from ref. 66 with permission from Springer Nature, copyright 2019. Reproduced from ref. 65 with permission from the American Chemical Society, copyright 2012.

The multilayered morphology of Ti_3_C_2_T_*x*_ is advantageous for charge storage applications. Its extensive surface area and open structural configuration create an optimal environment for ion transport and adsorption, which occur through both non-faradaic and faradaic mechanisms (Fig. 5a). At the surfaces of the MXene, electrostatic storage is facilitated by the reversible adsorption and desorption of ions *via* a faradaic charge-transfer mechanism. In this context, ions are initially adsorbed electrochemically onto the surface of MXene and subsequently diffuse through interlayer gaps and ion-conducting channels.^63,64^ Furthermore, the presence of numerous functional groups on MXene enhances its electrocatalytic activity across both basal and edge planes. Specifically, Ti_3_C_2_F_*x*_ MXene is well-suited for the oxygen evolution reaction due to its ability to adsorb active O_2_, while Ti_3_C_2_O_*x*_ MXene is regarded as advantageous for the hydrogen evolution and CO_2_ reduction reactions.^64^ Based on findings derived from DFT calculations, it has been established that a coulombic force is produced when functionalized Ti_3_C_2_T_*x*_ interacts with adsorbed Li^+^ ions at the carbon atoms during the initial phase of adsorption.^65^ Following this interaction, the lithiation process advances along the most efficient pathway, which is defined by the lowest energy barrier, thereby enhancing the capacity for Li^+^ ions (Fig. 5b).

Wei *et al.* prepared hollow Ti_3_C_2_T_*x*_ MXene by applying it onto the surface of poly(methyl methacrylate) (PMMA) nanospheres for the purpose of facilitating vanadium redox reactions.^67^ The heterostructure was then heated to prepare hollow MXene spheres, which were decorated into graphite-felt electrodes by dipping. The prepared electrodes were tested in vanadium redox flow batteries (VRFBs) to investigate their electrocatalytic properties using cyclic voltammetry, which were better than those observed for the pure carbon NP-based materials. At a high current density (300 mA cm^−2^), the electrolyte utilization efficiency was 62.9% and the energy efficiency was 75.0%, respectively. Interestingly, the battery displayed good stability and low energy efficiency decay at a current density of 200 mA cm^−2^ over 500 cycles. The excellent performance towards the V^3+^ and VO^2+^ redox reactions were due to its high electrical conductivity, flexibility, and chemical stability with natural hydrophilicity of the composites.^67^

The unique properties and intrinsic morphology metallic Ti_3_C_2_T_*x*_ of make it a reasonable choice for the manufacture of electrodes, which could be suitable for use in the field of energy storage applications. The dilemma in MXene commercialization is adaptation for large-scale industry, owing to the toxicity of the etching process and the harsh chemical conditions for its synthesis. At present, it seems that using the spark plasma sintering method to produce the MAX phase is inherently a batch process. The concerns about the etching procedure using HF should be investigated to control the particle size, defects, and toxicity.^37^

## 3. Applications of MXene-based functional materials

**Fig. 6:**
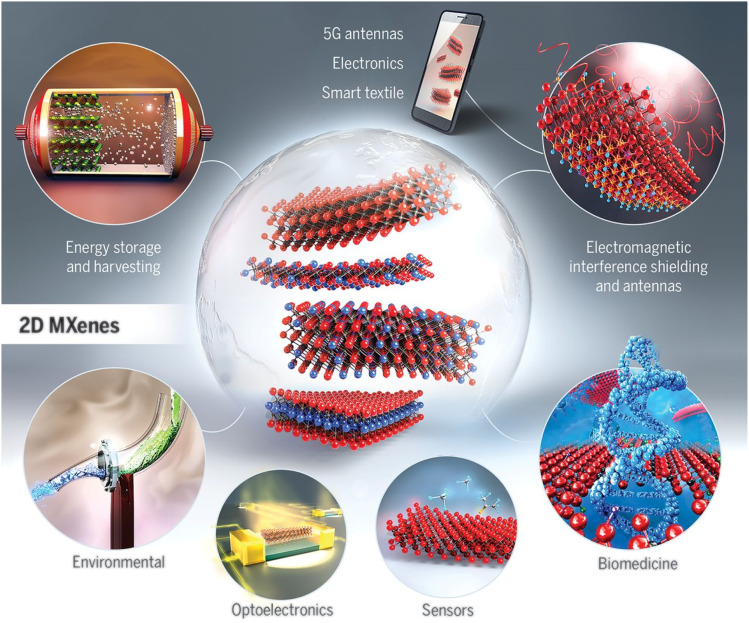
Structure and potential applications of 2D MXenes. Reproduced from ref. 68 with permission from the American Association for the Advancement of Science, copyright 2021.

MXene-based materials offer a wide range of potential applications owing to their unique properties. These applications span many fields, including but not limited to sensor, electrochemical, optical, electronics, biomedical, and energy applications, as summarized in Fig. 6. In this section, their applications in energy and environment will be investigated in detail.

### 3.1. Electrochemical energy storage

#### 3.1.1 Active and non-active materials

Ti_3_C_2_T_*x*_ MXenes have mainly been used in supercapacitors (SCs) and lithium-ion and sodium-ion batteries (SIB). Benefiting from their high specific area and abundant functional groups, the theoretical gravimetric capacity (TGC) of Ti_3_C_2_T_*x*_ MXenes was calculated to be around 268, 67, and 130 mA h g^−1^ for O-terminated, OH-terminated, and F-terminated materials, respectively.^69^

**Fig. 7:**
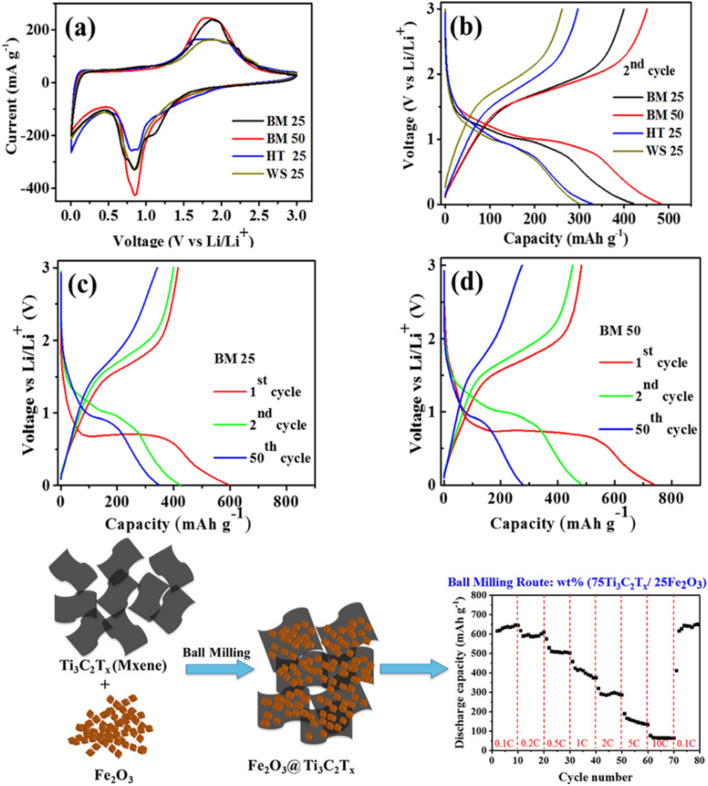
(a–d) Effect of preparation on the performance of Fe_2_O_3_/Ti_3_C_2_T_*x*_ anode materials for LIBs. Reproduced from ref. 71 with permission from American Chemical Society, copyright 2018.

Du *et al.* investigated the use of an FeS_2_@MXene composite for lithium and sodium ion storage, which demonstrated remarkable rate capabilities. Their findings indicate that its specific capacity for lithium-ion storage is approximately 762 mA h g^−1^ at a current density of 10 A g^−1^, whereas the specific capacity for sodium-ion storage is around 563 mA h g^−1^ at a current density of 0.1 A g^−1^.^70^ Ali *et al.* studied Fe_2_O_3_/Ti_3_C_2_T_*x*_ anode materials for LIBs.^71^ Hybrids were synthesized by confining Fe_2_O_3_ NPs in Ti_3_C_2_T_*x*_ in different mixing ratios *via* a dry ball-milling system, and the resulting heterostructures showed high surface areas. The optimized composite with 50 wt% Fe_2_O_3_ displayed the highest performance and stability (270 mA h g^−1^ at 1 C). The nanocomposites synthesized by the ball-milling method exhibited uniform distribution, a more accessible surface, and minimum restacking and oxidation of the nanosheets, which increased their electrochemical performance (Fig. 7). Moreover, excellent volumetric capacitances were recorded for a Ti_3_C_2_T_*x*_-based PVA hybrid in the electrolyte KOH, with values of 306 F Cm^−3^ and 528 F Cm^−3^ at 100 mV s^−1^ and 2 mV s^−1^, respectively.^61^ The use of the as-prepared composite with different electrolytes may widen its applications in the battery field.

Gentile and co-workers reported that Ti_3_C_2_T_*x*_ MXenes were synthesized in high-concentration HF and after post-synthesis 300 °C thermal treatments could be used as an anode in sodium-ion batteries (SIBs) with good rate capability and outstanding stability over 360 cycles. The pure Ti_3_AlC_2_ MAX phase was synthesized using spark plasma sintering.^41^ In fact, a lower etching rate offered better structural order to accelerate the electrochemical process. The OH-rich and H-rich compounds have higher insertion–deinsertion potential and smaller capacitive contributions as compared to those rich in –F terminal groups.^37^

Ti_3_C_2_T_*x*_ MXene has massively lower capacity (115 mA h g^−1^) but superior capacity retention (100% at 500 cycles) and better rate capability (90 mA h g^−1^ at 1.0 A g^−1^) compared to SoA hard carbons (300 mA h g^−1^, 85 mA h g^−1^ at 1.5 A g^−1^, and 65% at 500 cycles), respectively.^72^ Ti_3_C_2_T_*x*_ MXene-based supercapacitors are commonly asymmetric devices with negative electrodes as layered structures owing to MXene being oxidized at high potentials (>0.6 V *vs.* SHE).^45^

**Fig. 8:**
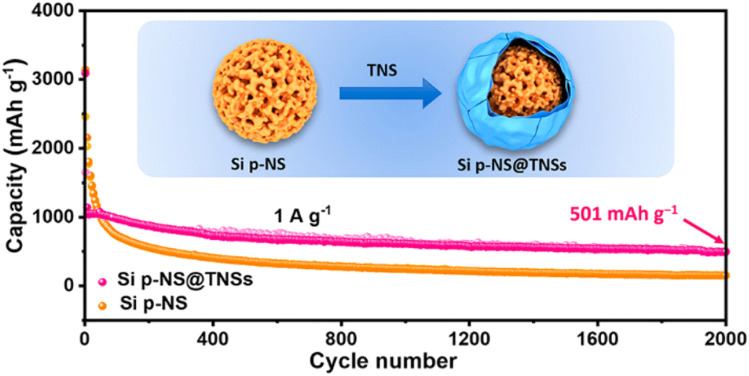
Interfacial assembly Ti_3_C_2_T_*x*_/Si for the enhancement of electrochemical Li storage activity. Reproduced from ref. 73 with permission from the American Chemical Society, copyright 2020.

In general, next-generation LIBs have utilized Si-rich composites as high-capacity anodes. However, Si has an excellent theoretical capacity but poor mechanical properties. To address this dilemma, the combination of Si and mesoporous carbon materials such as MXene enables the creation of a stable SEI. Xia and colleagues reported the preparation of a Si-based anode material in which Si *p*-NSs are wrapped with Ti_3_C_2_T_*x*_ MXene *via* an interfacial assembly method, as shown in Fig. 8.^73^ In the Si@Ti_3_C_2_T_*x*_ composite, the Ti_3_C_2_T_*x*_ MXene is characterized by an abundance of surface-terminating functional groups, which promotes robust interfacial interactions with the Si components, thereby enhancing the pseudocapacitive behavior and ensuring stable lithium storage. This interfacial synergy not only facilitates improved charge transfer kinetics, but also accommodates the volumetric changes that silicon undergoes during the lithiation and delithiation processes. Electrochemical characterization of the Si@Ti_3_C_2_T_*x*_ composite in a half-cell configuration revealed a notable reversible capacity of 1154 mA h g^−1^ after 150 cycles at a current density of 0.2 A g^−1^, accompanied by a remarkably low capacity decay rate of 0.026% per cycle. Additionally, the composite demonstrated exceptional long-term cycling stability, maintaining a capacity of 501 mA h g^−1^ over 2000 cycles at a current density of 1 A g^−1^. Comparison with other results indicates that the performances of the Si@Ti_3_C_2_T_*x*_ composites are in line with other carbon-based composites, providing evidence that Ti_3_C_2_T_*x*_ MXenes can be utilized to encapsulate Si and for other high-capacity conversion composite anodes.^73–75^

**Fig. 9:**
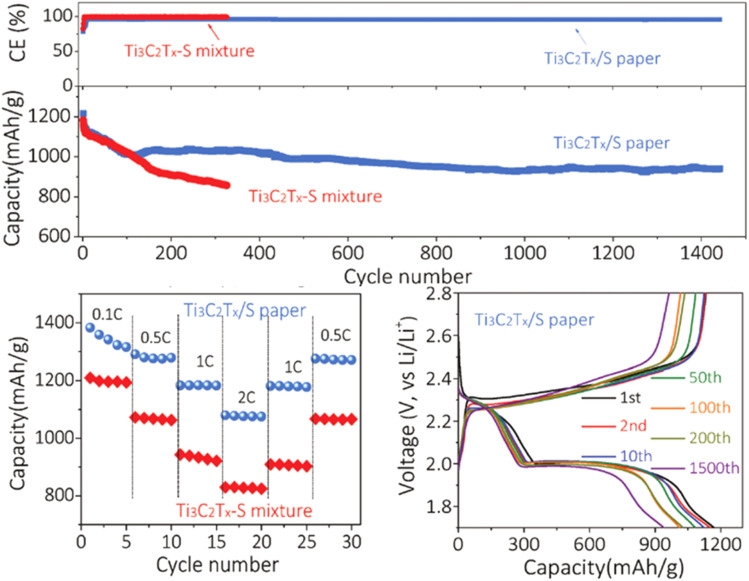
Cycling properties, rate capability, and charge–discharge profiles of the S@Ti_3_C_2_T_*x*_ composite. Reproduced from ref. 77 with permission from John Wiley and Sons, copyright 2019.

The Li–S system is one of the most critical systems in secondary batteries for next-generation electronics. Benefiting from the good dissolution of lithium polysulfide (LiPS) in the electrolyte, it avoids irreversible reactions that affect the cell integrity.^76^ Tang *et al.* synthesized a robust S@Ti_3_C_2_T_*x*_ composite combining LiPSS2 from LiF–HCl etched Ti_3_C_2_T_*x*_ MXene (Fig. 9). The optimized S@Ti_3_C_2_T_*x*_ composite displayed a uniform distribution of S in the characteristic multilayered Ti_3_C_2_T_*x*_ and had good electrochemical performance with ultralow capacity decay (0.014% after 1500 cycles) compared to pure S and Ti_3_C_2_T_*x*_.^77^

**Fig. 10:**
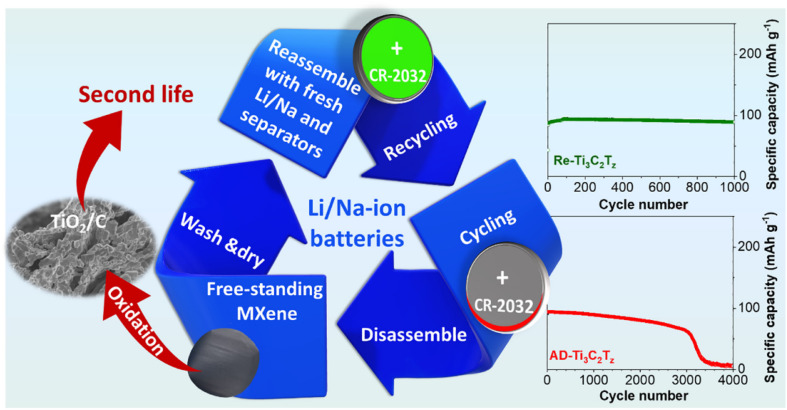
Second life of Ti_3_C_2_T_*x*_ electrodes for LIBs/SIBs. Reproduced from ref. 78 with permission from Elsevier, copyright 2023.

Ti_3_C_2_T_*x*_ from spent batteries was utilized as recycled electrodes for SIBs/LIBs by Li *et al.*^78^ In their work, free-standing delaminated Ti_3_C_2_T_*x*_ electrodes were synthesized *via* a vacuum system using TMAOH solutions and annealed at high temperature to give the anode material. The free-standing annealed delaminated-Ti_3_C_2_T_*x*_ nanostructures displayed much better electrochemical properties than those of delaminated Ti_3_C_2_T_*x*_ samples owing to the elimination of functional groups and surface water molecules. The annealed delaminated-Ti_3_C_2_T_*x*_ electrodes displayed superior cycling stability at 1 A g^−1^ after 2000 cycles with a capacity retention of 93% (Fig. 10). The recycling process avoids the pyrometallurgical procedure typically used in current battery recycling. The product after heat treatment under a CO_2_ atmosphere is TiO_2_/C, which can be used in the fields of electrochemical oxygen or photocatalytic hydrogen evolution and photodegradation.

In addition to the active material, the separator is pivotal in improving the electrochemical performance of Li–S batteries. Research conducted by Yang and colleagues indicated that MXene-based composites effectively regulate polysulfide shuttling and maintain stability at elevated temperatures. MXene nanosheets with an average size of less than 5 nm were synthesized through a hydrothermal method following an etching process and subsequently incorporated onto g-C_3_N_4_ to serve as a functional separator layer in Li–S batteries. This configuration achieved a remarkable specific capacity of 1433 mA h g^−1^, accompanied by an exceptionally low capacity decay rate of 0.024% per cycle at a rate of 2 C over 1000 cycles. The enhanced electrochemical activity can be attributed to the abundant active sites present on the MXene, in conjunction with the pyridinic-N structure of g-C_3_N_4_.^23,79^

#### 3.1.2 Anode and cathode

The electrochemical performance of Ti_3_C_2_T_*x*_ MXenes is limited due to their tendency to agglomerate or aggregate, which prevents ion movement along with electrolyte infiltration. In order to use MXene as an anode for LIBs, a 3D MXene with abundant active sites was prepared by a sulfur-template technique, and was flexible and freestanding.^80^ The porous MXene foam improves lithium storage capacity with excellent rate performance and an ultra-long-term cycle stability of 101 mA h g^−1^ at 18 A g^−1^ and 350 cycles, respectively.

**Fig. 11:**
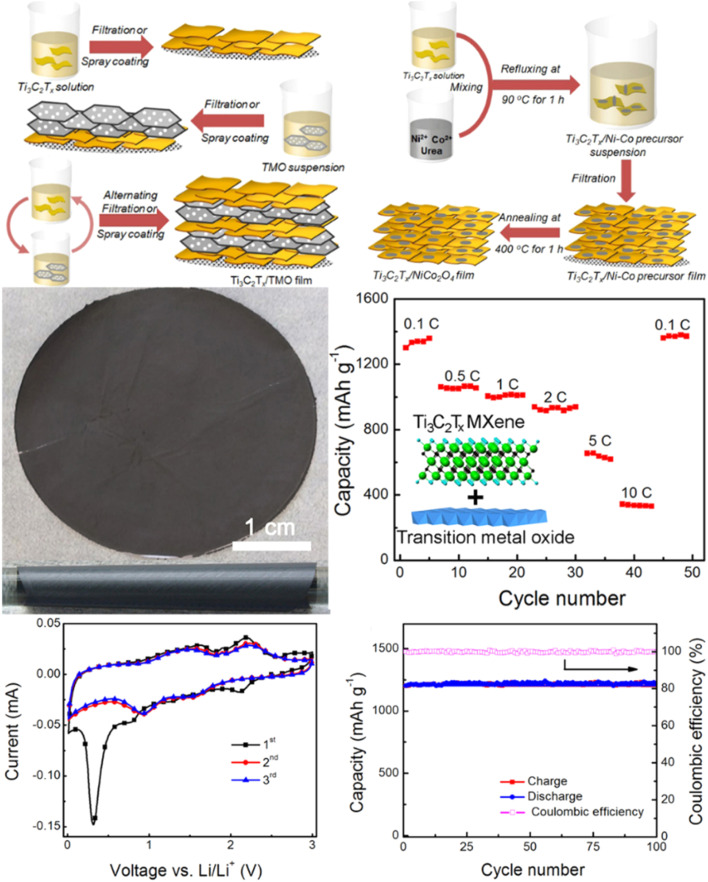
Schematic illustration of the combination of Ti_3_C_2_T_*x*_ MXene with transition metal oxide to obtain hybrid electrodes for energy storage. Reproduced from ref. 81 with permission from Elsevier, copyright 2016.

Gogotsi and co-coworkers synthesized flexible and conductive Ti_3_C_2_T_*x*_/Co_3_O_4_ and Ti_3_C_2_T_*x*_/NiCo_2_O_4_ composites for Li-ion storage by combining NiCo_2_O_4_ and Co_3_O_4_ with MXene using an alternating filtration method.^81^ As shown in Fig. 11, the hybrid film displayed an excellent reversible capacity of 1330 mA h g^−1^ at 0.1 C, along with enhanced rate capacity. The excellent electrical performance of the hybrid can be explained by the good metallic conductivity of MXene and the high theoretical capacity, good chemical stability, and low cost of Co_3_O_4_ nanoparticles, while ternary NiCo_2_O_4_ has two cations with good electrical conductivity. Table 1 presents a compilation of recent achievements in various MXene-based composites, along with their electrochemical performances.

**Table 1:** Comparison of MXene and its composites for energy storage applications

Material	Synthesis method	Electrochemical performance (capacitance)	Ref.
Ti_3_C_2_T_*x*_ MXene	HF etching	75 F g^−1^ @ 2 A g^−1^ (3 M Na_2_SO_4_)	96
MXene/MoSe_2_	LiF/HCl etching	183 mA h g^−1^@1 A g^−1^	97
MXene/graphene	Ultrasonic treatment	405 F g^−1^ (6 M KOH)	98
MXene/CoF	HF etching	1268 F g^−1^@1 A g^−1^ (0.1 M KOH)	99
MXene/Nb_2_C	Chemical etching	53 F g^−1^@0.3 A g^−1^ (1 M PVA/H_2_SO_4_)	100
MXene/CNT/PANI	*In situ* polymerization and physical assembly	429.4 F g^−1^@1 A g^−1^ (1.0 M H_2_SO_4_)	101
MXene/MnO_2_	Mild chemical deposition method	130.5 F g^−1^ @ 0.2 A g^−1^ (1 M Na_2_SO_4_)	102
MXene/BCN	Pyrolysis	245 F g^−1^@1 A g^−1^ (1 M PVA/H_2_SO_4_)	103
MXene/PEDOT:PSS	Solution-blending filtration	286 F g^−1^@2 mV s^−1^ (1 M H_2_SO_4_)	104
MXene/PANI@rGO	Solution etching	45 F g^−1^ (PVA–PAA–NHS)	105
MXene/heteroatom-doped N	Polishing method	390 F g^−1^ at 1 A g^−1^ (1 M H_2_SO_4_)	106
MXene/graphene@Ni	LiF/HCl etching	254 F g^−1^@1 A g^−1^	107
MXene/BC@PPy	Vacuum-filtration	290 mF cm^−2^	108

Another study investigated the self-assembly of SnO_2_ nanowires on Ti_3_C_2_T_*x*_ nanosheets for fast energy storage *via* van der Waals interactions.^82^ The as-synthesized SnO_2_/Ti_3_C_2_T_*x*_ composite could avert the agglomeration of the SnO_2_ nanowires during the lithiation/delithiation process and prevent the active sites from being lost, which provided short Li^+^ diffusion pathways. The chemical reaction between Li/Li^+^ and SnO_2_/Ti_3_C_2_T_*x*_ can be summarized briefly as follows:SnO_2_ + 4Li^+^ + 4e^−^ ↔ Sn + 2Li_2_OSn + *z*Li^+^ + ze^−^ ↔ Li_*z*_SnTi_3_C_2_T_*x*_ + *z*Li^+^ + *z*e^−^ ↔ Ti_3_C_2_T_*x*_Li_*z*_

Regarding the cathode, Ti_3_C_2_T_*x*_ MXenes have been extensively investigated as promising materials to enhance battery performance. An MXene/MoS_2_ composite cathode material was synthesized *via* a solvothermal approach and exhibited remarkable electrochemical performance. The Al/MXene/MoS_2_ battery demonstrated an initial capacity of 224 mA h g^−1^, which was maintained at 166 mA h g^−1^ after cycling. This performance is approximately 2.5 times greater than that of the Al/MoS_2_ battery, which recorded a capacity of 88 mA h g^−1^.^83^ The superior performance of the MXene/MoS_2_ composite is attributed to its significantly lower charge transfer resistance in comparison to the pure MoS_2_ cathode. The Ti_3_C_2_T_*x*_ MXene acts as a robust supporting framework, enhancing structural stability and reducing the pulverization of the MoS_2_ nanoflowers during the charge–discharge cycles. As anticipated, the MXene/MoS_2_ composite cathodes exhibited markedly lower charge transfer resistance and improved capacity retention compared to the MoS_2_-only cathodes. Additionally, the overlapping interlayer structure formed between the MXene multilayers and MoS_2_ nanoflowers increases the contact area, thereby facilitating enhanced electronic transport and further reducing charge transfer resistance.

Li and co-workers employed cetyltrimethylammonium bromide to expand the MXene interlayer spacing, followed by a selenization process to synthesize the composite cathode CTAB@Se/MXene. In aluminum-based batteries, the CTAB@Se/MXene composite demonstrated a high reversible specific discharge capacity of 583 mA h g^−1^ at a current density of 100 mA g^−1^.^84^ On the anode side, Al_2_Cl_7_^−^ ions decompose into metallic Al and AlCl_4_^−^. During the charging process, oxidation reactions associated with the Ti^2+^/Ti^3+^ and Ti^2+^/Ti^4+^ redox couples in MXene take place on the cathode side, accompanied by the insertion of AlCl_4_^−^ anions, as follows.

Anode:4Al_2_Cl_7_^−^ ↔ 7AlCl_4_^−^ + Al

Cathode:Ti_3_C_2_T_*x*_ + AlCl_4_^−^ ↔ Ti_3_(AlCl_4_)C_2_T_*x*_*y*Se + 2*z*AlCl_4_^−^ ↔ Se_*y*_Cl_*z*_ + *z*Al_2_Cl_7_^−^

#### 3.1.3 Triboelectric nanogenerators (TENGs)

Since the introduction of TENGs, there has been a significant surge of interest in their potential applications and the impact they could have on our daily lives. TENGs offer several advantages over traditional energy-harvesting technologies, such as ease of fabrication, cost-effectiveness, and the ability to convert low-frequency mechanical energy into electricity. These characteristics make TENGs a promising solution for powering small electronic devices, sensors, and even wearable technology.

Recently, the incorporation of MXene materials into TENGs has attracted increasing attention due to the unique properties of MXenes. These materials exhibit excellent electrical conductivity, mechanical flexibility, and surface functionalization capabilities, enhancing the overall performance of TENGs. For example, Cao *et al.* proposed an MXene liquid electrode to fabricate a stretchable and shape-adaptive TENG.^85^ In their study, the output voltage of the MXene-based TENGs reached up to 300 V. They highlighted the fact that the excellent fluidity and high electronegativity of the MXene liquid electrode provided the TENG with long-term reliability and stable electrical output.

Similarly, Du *et al.* demonstrated that the high electronegativity of MXenes effectively enhances the output performance of MXene-based TENGs.^86^ Their research presented an ultra-flexible and self-healable TENG with highly efficient electromagnetic interference shielding composed of modified Ti_3_C_2_T_*x*_ MXene (*m*-MXene)-based nanocomposite elastomers. Benefiting from the excellent electronegativity of *m*-MXene, the single-electrode TENG generated a high open-circuit voltage (*V*_oc_) ranging from −65 to 245 V, a short-circuit current (*I*_sc_) of 29 μA, and a peak power density of 1150 mW m^−2^, and was capable of powering twenty light-emitting diodes (LEDs).

Cai *et al.* explored the effect of surface chemistry on the work function of MXenes, which determines the performance of MXene-based TENGs.^87^ Their first-principles calculations revealed that surface functional groups significantly influence the work function of MXenes: –OH termination reduces the work function compared to a bare surface, while –F and –Cl increase it. Due to these exceptional properties, MXenes have been used as additives in TENGs *via* doping or blending methods. For instance, Luo *et al.* reported that MXene nanosheet doping promoted the crosslinking of a PVA hydrogel, improving its stretchability.^88^ The MXene nanosheets also formed microchannels on the surface, enhancing the conductivity of the hydrogel by improving ion transport and generating an additional triboelectric output *via* a streaming vibration potential mechanism.

**Fig. 12:**
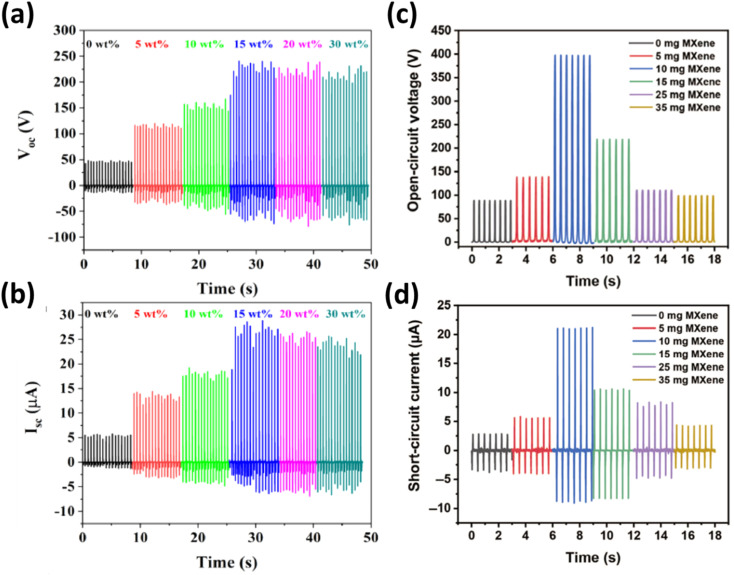
Output performance of the as-fabricated TENG: (a) open-circuit voltage; (b) short-circuit current. Output performance of the crumpled MXene-based TENG: (c) open circuit voltage; (d) short circuit voltage. Reproduced from ref. 94 with permission from Springer Nature, copyright 2021. Reproduced from ref. 95 with permission from Elsevier, copyright 2022.

Similarly, Gao's research illustrated that MXene doping enhanced the crystallinity of the composite films, resulting in a 450% improvement in tensile properties and an 80% reduction in wear volume during friction tests.^89^ The as-fabricated TENG using this composite film produced an open-circuit voltage of 397 V, a short-circuit current of 21 μA, and a transfer charge quantity of 232 nC, which were 4, 6, and 6 times higher, respectively, than those of a TENG made with pure PTFE film, as depicted in Fig. 12a and b. This work provided an innovative strategy to simultaneously improve the mechanical and electrical properties of TENGs.

MXenes have also been shown to enhance dielectric properties and surface charge density. Bhatta *et al.* found that blending Ti_3_C_2_T_*x*_ nanosheets into a PVDF matrix substantially improved triboelectric performance.^90^ The dielectric modulation of PVDF nanofibers by incorporating conductive MXene nanosheets increased the dielectric constant by 270% and the surface charge density by 80%. Mirsepah *et al.* further demonstrated that MXene integration improved TENG performance.^91^ To prepare stretchable MXene-based triboelectric layers without compromising triboelectric properties, one approach involves compositing MXene with inherently stretchable materials. However, this method can lead to disadvantages, such as reduced electrical conductivity, limited stretchability, and slow response to external stimuli, limiting practical applications.

To address these issues, Cao *et al.* introduced a stretchable TENG using crumpled MXene films created by brush-coating MXene ink onto a pre-stretched latex substrate, followed by release.^92^ Additionally, Answer *et al.* incorporated a thin film of micron-sized ultrathin Ti_3_C_2_T_*x*_ MXene sheets (TMSs) into a polyethylene terephthalate (PET)-based tribo-negative electrode.^93^ After optimizing both triboelectric layers, the TMS-TENG achieved an open-circuit voltage of ∼390 V, a short-circuit current (*I*_sc_) of ∼96 μA, and a power density of 6.66 W m^−2^, as displayed in Fig. 12c and d.

In comparison to alternative materials for energy storage, MXenes with surface functional groups exhibit several beneficial characteristics, including high electrical conductivity and a layered structure that promotes swift ion intercalation and deintercalation. Their remarkable mechanical flexibility and hydrophilicity, coupled with the presence of titanium, contribute to pseudocapacitive behavior through the availability of numerous redox-active sites. However, several challenges must be addressed to fully exploit their capabilities. These challenges include the propensity for restacking due to van der Waals interactions, as well as oxidation instability in humid and aqueous environments. Furthermore, the synthesis process, which frequently involves hazardous and complex methods such as HF etching, presents obstacles regarding scalability and safety. Lastly, careful control of ion selectivity and compatibility across various electrolytes is essential to ensure optimal performance.

### 3.2. Water remediation

The application of Ti_3_C_2_T_*x*_ for environmental treatment and resource recovery has been of utmost importance in this field of research. In this section, the fundamentals of the removal of various pollutant *via* the utilization of Ti_3_C_2_T_*x*_ are addressed. The areas of focus are heavy metal ions in wastewater, dye degradation, and radionuclides.

#### 3.2.1 Desalination applications

**Fig. 13:**
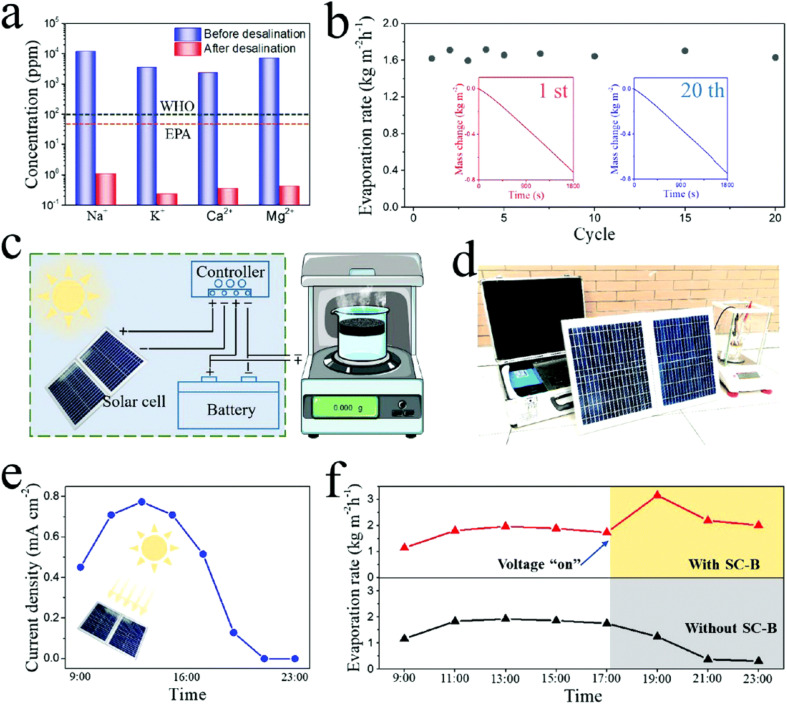
(a) Water quality before and after evaporation desalination. (b) Evaporation durability activity over 1 cycle and 20 cycles. (c and d) Schematic and optical photograph of the steam generation system. (e) Charging current density of the system from morning to night. (f) Water evaporation rate over one day. Reproduced from ref. 109 with permission from the Royal Society of Chemistry, copyright 2020.

Pure Ti_3_C_2_T_*x*_ MXenes and their heterostructures are potential competitors for desalination applications owing to their properties such as high surface area, good mechanical properties, excellent hydrophilicity, and long-duration stability with a low contact angle (21.5°) of water on their surface. For example, Zhao *et al.* reported that Ti_3_C_2_T_*x*_-based aerogels can work well for electro-thermal and photo-thermal conversion with high efficiency.^109^ The authors designed a steam generation system that includes macroscopic Ti_3_C_2_T_*x*_ architectures interconnected with a solar cell battery in both sunny and dark conditions, resulting in a high evaporation rate (1.62 kg m^−2^ h^−1^ with a 2.5 V voltage supply) and providing 14 h of operation per day. The designed system converts sunlight into electricity and stores it in a battery on sunny days for power for the composites to generate steam, which reduces extra electricity consumption (Fig. 13).

Tan and co-workers studied a Ti_3_C_2_T_*x*_ coating that improved the photothermal performance and fouling-resistance of a PVDF membrane in solar-assisted membrane distillation.^110^ The photothermal conversion was calculated to be 5.8 kW m^−2^, and after 21 h, the PVDF/Ti_3_C_2_T_*x*_ composite conferred a reduction of around 65% in flux decline in comparison with the uncoated membrane. The as-synthesized composite was able to prevent protein fouling and offer localized heating under light illumination with a large surface area from the multilayered structures.^111,112^ The photocatalytic performance can endow the photothermal membrane with self-cleaning functionality.

Li *et al.* synthesized biomimetic MoS_2_/GO/Ti_3_C_2_T_*x*_ nanocoatings with improved light-to-heat conversion (up to 93.2%) for solar steam generation.^113^ The bioinspired Ti_3_C_2_T_*x*_ nanocoatings resulted in a small loading of solar thermal composite (around 0.32 mg cm^−2^) but guaranteed high efficiency (1.33 kg m^−2^ h^−1^) as compared to another state-of-the-art device.

#### 3.2.2 Radionuclide elimination

In recent years, several studies have reported that Ti_3_C_2_T_*x*_ MXene acts as an adsorbent for the elimination of radionuclides, specifically, uranium (U(vi)), europium III (Eu(iii)), thorium (Th(iv)), and cesium (Cs^+^). The main mechanisms of radionuclide elimination are the adsorptive mechanism and electrostatic interaction. The surface terminations of Ti_3_C_2_T_*x*_ MXene are negatively charged, which is beneficial to adsorb cations.

**Fig. 14:**
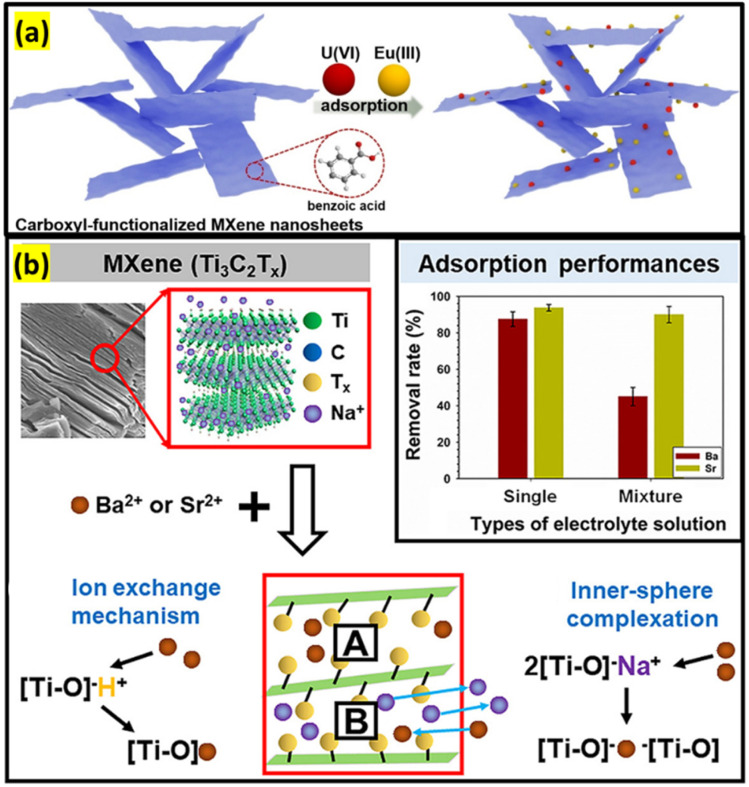
Carboxyl-functionalized Ti_3_C_2_T_*x*_ nanosheets (a) for the removal of U(vi) and Eu(iii), and (b) for treatment of Ba^2+^ and Sr^2+^. Reproduced from ref. 114 and 115 with permission from Elsevier, copyright 2020.

Zhang and co-workers revealed that carboxyl-terminated Ti_3_C_2_T_*x*_ MXene displays excellent removal capability for Eu(iii) and U(vi) with high adsorption ability (345 mg g^−1^ for U and 97 mg g^−1^ for Eu).^114^ The aryl diazonium salt plays an important role in the stability of the catalyst in water after a one-week stability test, preventing the oxidation process of raw MXene. The key mechanism for improving the removal of radionuclide ions on the composite is the strong affinity of UO^2+^ and Eu^2+^ coordinated with the carboxyl terminations, creating inner-sphere surface complexes. Moreover, ion exchange and electrostatic interaction also partially contributed to the effective enrichment of radionuclides. In the same context, inner-sphere complex formation and chemical ion exchange properties are dominant in the adsorption of Ba^2+^/Sr^2+^ by Ti_3_C_2_T_*x*_ MXene.^115^ Since the electronegativity of Sr^2+^ (1.0) is greater than that of Ba^2+^ (0.9), Ba^2+^ tends to react with the negative surface charges of Ti_3_C_2_T_*x*_ (Fig. 14). The Ti_3_C_2_T_*x*_ surface charge becomes more negative with increasing pH value, which improves the free energy between adsorbent and adsorbates. Therefore, the Ti_3_C_2_T_*x*_-based catalysts have potential in water purification of model fracking wastewater and radioactive wastewater.

#### 3.2.3 Removal of organic dyes

Currently, there are more than ten thousand different organic dyes on the market, which are challenging pollutants to treat owing to their sophisticated molecular structure and chemical stability.^116^ It is believed that some organic dyes including phthalocyanine dyes or metal-associated dyes are DNA mutagenic and cancer-causing due their aromatic molecular structure. The discharge of dyes into natural water resources leads to damage to natural life, aquatic ecosystems, and even the renal system of animals.^117,118^ The specific properties of dyes, such as high thermal- and photostability, could be used to develop physical treatments involving utilization of light or temperature, or even using conventional aerobic or anaerobic biological techniques. Additionally, the treatment of dye-contaminated wastewater has been studied using a passive uptake method employing bio-sorbents.^117^

**Fig. 15:**
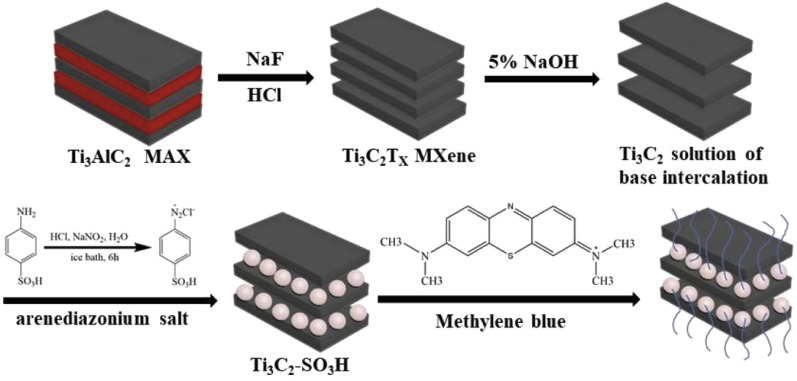
Ti_3_C_2_T_*x*_–SO_3_H composite for the removal of methylene blue in an alkaline environment. Reproduced from ref. 120 with permission from Elsevier, copyright 2019.

With the economic drawbacks associated with traditional adsorbents, metallic MXene represents a suitable candidate for treating organic-dye-polluted wastewater. Research into the potential application of Ti_3_C_2_T_*x*_ MXenes for the removal of organic dyes such as methylene blue, 2,4-dinitrophenol, and rhodamine B has been reported based on their better adsorption than several other 2D materials and conventional adsorbents. In order to improve the effectiveness of MXenes, combination and functionalization through the grafting method were considered. The main mechanism of the interaction of Ti_3_C_2_T_*x*_ MXenes with pollutants has been reported to be single-layer based on the Freundlich and Langmuir isotherms.^119,120^ As shown in Fig. 15, a Ti_3_C_2_T_*x*_–SO_3_H adsorbent was prepared by coupling-diazotization, and the adsorbent exhibited efficient removal of the cationic dye MB (111.11 mg g^−1^).^120^ The positive enthalpy changes (Δ*H*^0^; J mol^−1^) indicated that the adsorption of MB onto the catalysts is an endothermic process, while the negative Gibbs energy changes (Δ*G*^0^; J mol^−1^) demonstrated that the reaction was spontaneous. Moreover, the electrostatic interaction plays an essential role in the removal of methylene blue.

The limitations of Ti_3_C_2_T_*x*_ MXenes for dye removal are their instability under air, CO_2_, and other environments. Moreover, a comprehensive assessment of the toxicity of Ti_3_C_2_T_*x*_ on humans and other organisms has not yet been performed, which limits the application of Ti_3_C_2_T_*x*_ in dye removal.^119^ Surface modification is an effective method for improving the biocompatibility of Ti_3_C_2_T_*x*_ and diminishing its cytotoxic effects on natural ecosystems. MXenes have been modified with dopamine, polyethylene glycol, hyaluronic acid, and glucose to improve their durability.^121^ In fact, collagen-combined Ti_3_C_2_T_*x*_ displayed lower toxicity and good cell viability over A375 human skin (malignant melanoma cells) using a zeta potential analyzer.^122^ An analysis of toxicity *in vitro* indicated that the modification of Ti_3_C_2_T_*x*_ MXene with collagen decreases oxidative stress and the generation of reactive oxygen species in non-malignant cells. Gu *et al.* reported a comparison of the toxicity of MXene quantum dots at the same mass using human endothelial cells (HUVECs). The results confirmed that Ti_3_C_2_T_*x*_ MXenes are more toxic than Nb_2_CT_*x*_ MXene to HUVECs.^123^ The preparation procedure of Ti_3_C_2_T_*x*_ is still sophisticated and requires many reaction steps, hazardous acids, and specific precautions, and has a low production yield relative to the precursors, which prevent the scale-up of Ti_3_C_2_T_*x*_ for applied water treatment process. A summary the use of Ti_3_C_2_T_*x*_ MXenes and their composites for organic dye removal is provided in Table 2.

**Table 2:** Comparison of Ti_3_C_2_T_*x*_ MXene-based composites for organic dye removal in wastewater^a^

Pollutant	Composite	Uptake/efficiency	Mechanism	Ref.
MB	LiOH–Ti_3_C_2_T_*x*_	121 mg g^−1^	Adsorption	124
MB	Ti_3_C_2_T_*x*_	100 mg g^−1^	Adsorption	124
MB	NaOH–Ti_3_C_2_T_*x*_	189 mg g^−1^	Adsorption	124
MB	KOH–Ti_3_C_2_T_*x*_	77 mg g^−1^	Adsorption	124
ST	MXene-COOH@(PEI/PAA)_*n*_	33 mg g^−1^	Adsorption	125
NR	MXene-COOH@(PEI/PAA)_*n*_	42 mg g^−1^	Adsorption	125
MB	Ti_3_C_2_T_*x*_	140 mg g^−1^	Adsorption	126
AB	Ti_3_C_2_T_*x*_	200 mg g^−1^	Adsorption	126
MB	Phytic acid (PA)-Ti_3_C_2_T_*x*_	42 mg g^−1^	Adsorption	127
RhB	Phytic acid (PA)-Ti_3_C_2_T_*x*_	22 mg g^−1^	Adsorption	127
MB	Surface charged Ti_3_C_2_T_*x*_	2460 mg g^−1^	Adsorption	128
MB	F-terminated Ti_3_C_2_T_*x*_	92%	Adsorption	129
MB	h Ti_3_C_2_T_*x*_	24 mg g^−1^	Adsorption	130
MB	Ti_3_C_2_T_*x*_	39 mg g^−1^	Adsorption	131
MB	Cellulose ester/Ti_3_C_2_T_*x*_	100%	Adsorption	132
CR	PEI/Ti_3_C_2_T_*x*_	3568 mg g^−1^	Adsorption	133
MB	AAC/Ti_3_C_2_T_*x*_	311.5	Adsorption	134
MO	Ti_3_C_2_T_*x*_	94 mg g^−1^	Adsorption	135
Cr(vi)	Ti_3_C_2_T_*x*_	104 mg g^−1^	Adsorption	135
MB	Ti_3_C_2_T_*x*_–SO_3_H	111 mg g^−1^	Adsorption	120
MB	Ti_3_C_2_T_*x*_/sodium alginate	92 mg g^−1^	Adsorption	136
MB	Ti_3_C_2_T_*x*_/Fe_3_O_4_	1.71 mg g^−1^	Reduction/adsorption	137
RhB	Ti_3_C_2_T_*x*_/Co_3_O_4_	47 mg g^−1^	Reduction/adsorption	138
MB	Ti_3_C_2_T_*x*_/Co_3_O_4_	136 mg g^−1^	Reduction/adsorption	138

a MB: methylene blue, ST: safranine T, NR: neutral red, AB: acid blue 80, RhB: rhodamine B, CR: congo red, MO: methyl orange.

The multilayered morphology and extensive surface area of MXene-based materials significantly enhance pollutant adsorption by offering numerous active sites and interlayer spacing, which promote direct interactions with surface functional groups through mechanisms such as hydrogen bonding, electrostatic attraction, and chelation. Their superior electrical conductivity and highly reactive surfaces facilitate rapid adsorption kinetics and increased reaction rates through ion exchange and surface complexation. Nonetheless, a critical drawback of MXene-based materials is their susceptibility to oxidation when exposed to light and oxygen-rich environments, which can lead to restacking and aggregation. These processes considerably diminish the accessible surface area and the availability of active sites. Additionally, the potential environmental toxicity of MXenes necessitates comprehensive evaluation prior to their large-scale application and environmental release.

## 4. Conclusion and perspectives

This review summarizes the recent advances in the use of Ti_3_C_2_T_*x*_ MXene as a catalyst, adsorbent, and photocatalytic agent in energy storage and dye removal from wastewater. MXenes have been prepared using various etchants (HCl/LiF, NaF/LiF/KF), with a focus on direct HF and in-situ-formed HF etchants for effective products. After etching, organic molecules are associated with the intercalation and delamination of single-layered Ti_3_C_2_T_*x*_ MXene. Tip sonication offers a smaller flake size of Ti_3_C_2_T_*x*_ with defects relative to bath sonication, which should optimize the sonication parameters. Although successful results have been obtained, further in-depth exploration is required.

Regarding the raw sources, the MAX phase is prepared at high temperatures using sophisticated machine systems. Toxic HF and other fluoride-containing etchants have been used to etch Ti_3_C_2_T_*x*_ MXene; more efforts are needed in this particular aspect. Connections between theoretical modeling calculations and the practical applications of Ti_3_C_2_T_*x*_ need to be established to provide the fundamentals for understanding the unique composition-related properties of Ti_3_C_2_T_*x*_ MXenes. Subsequently, the scale-up process between laboratories and industries could be solved and avoid risks. From the point of view of multilayered structures, the lamellar structure of the MXene-based materials has a significant effect on their results as either sorbents or electrodes. Furthermore, engineering high-surface-area materials using Ti_3_C_2_T_*x*_ MXene-derived composites is massively desirable for enhanced performance. Finally, further research efforts should be devoted to applications. Although massive enhancements have been achieved, the performance stability of Ti_3_C_2_T_*x*_ MXene-derived composite sorbents and electrodes still need to surpass that of conventional carbon-based materials.

In wastewater treatment, it is essential to elucidate a comprehensive and plausible mechanism underlying the interaction between adsorbates and Ti_3_C_2_T_*x*_-based sorbents. This understanding is crucial for guiding the design and application of MXene materials to a broad range of contaminants. Despite employing similar raw materials and synthesis techniques, significant variations in sorption performance are often observed and achieved. These discrepancies underscore the need for a deeper investigation into the physicochemical interactions at the molecular level, including surface functional groups, interlayer spacing, and the role of terminal groups (–OH, –O, –F), which significantly influence adsorption capacity and selectivity.

For energy storage applications, the principal challenge in transitioning Ti_3_C_2_T_*x*_ MXene-derived composites from laboratory-scale research to commercial viability lies in the scalable production of materials with large surface areas, structural uniformity, and reproducible electrochemical performance. Overcoming synthesis-related inconsistencies, such as flake aggregation, oxidation during processing, and variability in surface terminations, will be crucial to ensuring consistent device performance. Moreover, the integration of Ti_3_C_2_T_*x*_ MXenes into TENG has emerged as a promising strategy to enhance energy conversion efficiency and expand the functionality of self-powered systems. This synergy has opened new opportunities in fields such as environmental sensing, wearable electronics, and sustainable energy harvesting. The unique combination of TENG technology with the tunable properties of MXenes represents a significant advancement, offering multifunctional platforms capable of simultaneously addressing energy and environmental challenges.

In conclusion, the application of Ti_3_C_2_T_*x*_ MXene in both environmental remediation and energy storage presents a transformative pathway to tackle pressing global issues such as energy depletion and water pollution. Although challenges remain in terms of scalability, stability, and mechanistic understanding, continued research efforts and innovative material design are well justified and hold substantial potential for real-world impact toward sustainable development in the 4.0 era.

## Conflicts of interest

The authors declare that they have no known competing financial interests or personal relationships that could have influenced the work reported in this study.

## Data Availability

No data were used in the research described in the article.

## References

[cit1] Tran M. N., Skorynina A., Addad A., Fadel A., Ben Tayeb K., Karmazin L., Thomas L., Corda M., Wisse Y., Vovk O., Khodakov A. Y., Grandidier B., Ordomsky V. (2025). Appl. Catal., B.

[cit2] Wu J., Yin G., Liu J., Yu Z. Z., Li X. (2025). Mater. Horiz..

[cit3] Malin S. A. (2025). Energy Res. Soc. Sci..

[cit4] Li C., Zhou D., Zheng F., Wang Y., Bi K. (2025). Chem. Eng. J..

[cit5] Felsmann B., Guerrini A., Hajdari G., Kis A., Romano G. (2025). Appl. Energy.

[cit6] Jägerskog A. , Clausen T. J., Holmgren T. and Lexén K., Energy and Water: The Vital Link for a Sustainable Future, Stockholm International Water Institute (SIWI), Stockholm, 2014

[cit7] Ma Q., Yu Y., Sindoro M., Fane A. G., Wang R., Zhang H. (2017). Adv. Mater..

[cit8] Goswami D. Y. and Kreith F., in Energy Conversion, CRC Press, 2017, pp. 1–30

[cit9] Panday A., Bansal H. O. (2014). Int. J. Global Energy.

[cit10] Ta Q. T. H., Cho E., Sreedhar A., Noh J.-S. (2019). J. Catal..

[cit11] Lee P. S., Chen X. (2014). Small.

[cit12] Gogotsi Y. , MXenes: From Discovery to Applications of Two-Dimensional Metal Carbides and Nitrides, CRC Press, 2023

[cit13] Ta Q. T. H., Thakur D., Noh J.-S. (2023). Chemosensors.

[cit14] Sreedhar A., Ta Q. T. H., Noh J.-S. (2022). Chemosphere.

[cit15] Eswaran S. G., Rashad M., Kumar A. S. K., Mahdy A. F. M. E. (2025). Chem.–Asian J..

[cit16] Ta Q. T. H., Tran N. M., Tri N. N., Sreedhar A., Noh J. S. (2021). Chem. Eng. J..

[cit17] Diedkova K., Pogrebnjak A. D., Kyrylenko S., Smyrnova K., V Buranich V., Horodek P., Zukowski P., Koltunowicz T. N., Galaszkiewicz P., Makashina K. (2023). ACS Appl. Mater. Interfaces.

[cit18] Anasori B., Gogotsi Y. (2023). Graphene 2D Nanomater..

[cit19] Alhabeb M., Maleski K., Anasori B., Lelyukh P., Clark L., Sin S. (2017). Chem. Mater..

[cit20] Praus P., Smýkalová A., Škuta R., Koštejn M., Pavlovský J., Tokarský J., Foniok K., Edelmannová M. F., Kočí K. (2023). J. Photochem. Photobiol., A.

[cit21] Nguyen T. P., Nguyen D. M. T., Le H. K., Vo D.-V. N., Lam S. S., Varma R. S., Shokouhimehr M., Nguyen C. C., Van Le Q. (2020). Mol. Catal..

[cit22] Phuong N.
T. T., Nguyen T. T., Nam N. N., Trinh K. T. L. (2023). Sens. Actuators, A.

[cit23] Yang K., Li C., Qi H., Dai Y., Cui Y., He Y. (2023). J. Mater. Chem. A.

[cit24] Cao Z., Zhu Y. B., Chen K., Wang Q., Li Y., Xing X., Ru J., Meng L. G., Shu J., Shpigel N., Chen L. F. (2024). Adv. Mater..

[cit25] Cao Z., Hu H., Ho D. (2022). Adv. Funct. Mater..

[cit26] Cao Z., Liang G., Ho D., Zhi C., Hu H. (2023). Adv. Funct. Mater..

[cit27] Zhang J., Wang K., Lu P., Gao J., Cao Z., Mo F., Ho D., Li B., Hu H. (2024). Adv. Funct. Mater..

[cit28] Ta Q. T. H., Nhiem L. T., Oanh D. T. Y., Hieu N. H., Nguyen P. K. T. (2024). Vietnam J. Chem..

[cit29] Hemanth N. R., Kim T., Kim B., Jadhav A. H., Lee K., Chaudhari N. K. (2021). Mater Chem Front.

[cit30] Chaudhari N. K., Hanuel Jin ab, Kim B., San Baek D., Hoon Joo S., Lee K. (2017). J. Mater. Chem. A.

[cit31] Sreedhar A., Ravi P., Noh J.-S. (2024). J. Mater. Sci. Technol..

[cit32] Gan Y., Xiong Y. (2025). RSC Adv..

[cit33] Zhang S., Wang L., Feng Z., Wang Z., Wang Y., Wei B., Liu H., Zhao W., Li J. (2025). ACS Nano.

[cit34] Mubeen I., Shah S., Pervaiz E., Miran W. (2024). Mater. Sci. Energy Technol..

[cit35] Ta Q. T. H., Thakur D., Noh J.-S. (2023). Chemosensors.

[cit36] Wang C., Tracy C. L., Ewing R. C. (2020). Appl. Phys. Rev..

[cit37] Ferrara C., Gentile A., Marchionna S., Ruffo R. (2021). Curr. Opin. Electrochem..

[cit38] Amara U., Hussain I., Ahmad M., Mahmood K., Zhang K. (2023). Small.

[cit39] Tran N. M., Ta Q. T. H., Noh J.-S. (2021). Appl. Surf. Sci..

[cit40] Benchakar M., Loupias L., Garnero C., Bilyk T., Morais C., Canaff C., Guignard N., Morisset S., Pazniak H., Hurand S. (2020). Appl. Surf. Sci..

[cit41] Gentile A., Ferrara C., Tosoni S., Balordi M., Marchionna S., Cernuschi F., Kim M., Lee H., Ruffo R. (2020). Small Methods.

[cit42] Li T., Yao L., Liu Q., Gu J., Luo R., Li J., Yan X., Wang W., Liu P., Chen B. (2018). Angew. Chem., Int. Ed..

[cit43] Wang Y., Zhang J., Wang X., Antonietti M., Li H. (2010). Angew. Chem., Int. Ed..

[cit44] Mahabari K., Mohili R. D., Patel M., Jadhav A. H., Lee K., Chaudhari N. K. (2024). Nanoscale Adv..

[cit45] Yang W., Yang J., Byun J. J., Moissinac F. P., Xu J., Haigh S. J., Domingos M., Bissett M. A., Dryfe R. A. W., Barg S. (2019). Adv. Mater..

[cit46] Prakash N. J., Kandasubramanian B. (2021). J. Alloys Compd..

[cit47] Ghidiu M., Lukatskaya M. R., Zhao M. Q., Gogotsi Y., Barsoum M. W. (2015). Nature.

[cit48] Jawaid A., Hassan A., Neher G., Nepal D., Pachter R., Kennedy W. J., Ramakrishnan S., Vaia R. A. (2021). ACS Nano.

[cit49] An Y., Tian Y., Shen H., Man Q., Xiong S., Feng J. (2023). Energy Environ. Sci..

[cit50] Pei Y., Zhang X., Hui Z., Zhou J., Huang X., Sun G., Huang W. (2021). ACS Nano.

[cit51] Liu J., Zhang H., Sun R., Liu Y., Liu Z., Zhou A., Yu Z. (2017). Adv. Mater..

[cit52] Lipatov A., Alhabeb M., Lukatskaya M. R., Boson A., Gogotsi Y., Sinitskii A. (2016). Adv. Electron. Mater..

[cit53] Kong F., He X., Liu Q., Qi X., Zheng Y., Wang R., Bai Y. (2018). Ceram. Int..

[cit54] Wang K., Zhou Y., Xu W., Huang D., Wang Z., Hong M. (2016). Ceram. Int..

[cit55] Li G., Jiang K., Zaman S., Xuan J., Wang Z., Geng F. (2019). Inorg. Chem..

[cit56] Naguib M., Mashtalir O., Lukatskaya M. R., Dyatkin B., Zhang C., Presser V., Gogotsi Y., Barsoum M. W. (2014). Chem. Commun..

[cit57] Xiong D., Li X., Bai Z., Lu S. (2018). Small.

[cit58] Borysiuk V. N., Mochalin V. N., Gogotsi Y. (2015). Nanotechnology.

[cit59] Lipatov A., Lu H., Alhabeb M., Anasori B., Gruverman A., Gogotsi Y., Sinitskii A. (2018). Sci. Adv..

[cit60] Naguib M., Kurtoglu M., Presser V., Lu J., Niu J., Heon M., Hultman L., Gogotsi Y., Barsoum M. W. (2011). Adv. Mater..

[cit61] Ling Z., Ren C. E., Zhao M.-Q., Yang J., Giammarco J. M., Qiu J., Barsoum M. W., Gogotsi Y. (2014). Proc. Natl. Acad. Sci. U. S. A..

[cit62] Liu H., Du C., Liao L., Zhang H., Zhou H., Zhou W., Ren T., Sun Z., Lu Y., Nie Z., Xu F., Zhu J., Huang W. (2022). Nat. Commun..

[cit63] Augustyn V., Come J., Lowe M. A., Kim J. W., Taberna P. L., Tolbert S. H., Abruña H. D., Simon P., Dunn B. (2013). Nat. Mater..

[cit64] Fei L., Lei L., Xu H., Guo X., Chen B., Han X., Chen X., Huang Q., Wang D. (2025). Carbon Energy.

[cit65] Tang Q., Zhou Z., Shen P. (2012). J. Am. Chem. Soc..

[cit66] Choi C., Ashby D. S., Butts D. M., DeBlock R. H., Wei Q., Lau J., Dunn B. (2019). Nat. Rev. Mater..

[cit67] Wei L., Xiong C., Jiang H. R., Fan X. Z., Zhao T. S. (2020). Energy Storage Mater..

[cit68] Mohammadi A. V., Rosen J., Gogotsi Y. (2021). Science.

[cit69] Zhang J., Kong N., Uzun S., Levitt A., Seyedin S., Lynch P. A., Qin S., Han M., Yang W., Liu J. (2020). Adv. Mater..

[cit70] Du C. F., Liang Q., Zheng Y., Luo Y., Mao H., Yan Q. (2018). ACS Appl. Mater. Interfaces.

[cit71] Ali A., Hantanasirisakul K., Abdala A., Urbankowski P., Zhao M.-Q., Anasori B., Gogotsi Y., Aïssa B., Mahmoud K. A. (2018). Langmuir.

[cit72] Bommier C., Luo W., Gao W.-Y., Greaney A., Ma S., Ji X. (2014). Carbon N Y.

[cit73] Xia M., Chen B., Gu F., Zu L., Xu M., Feng Y., Wang Z., Zhang H., Zhang C., Yang J. (2020). ACS Nano.

[cit74] Shen T., Xia X., Xie D., Yao Z., Zhong Y., Zhan J., Wang D., Wu J., Wang X., Tu J. (2017). J. Mater. Chem. A.

[cit75] Liu N., Lu Z., Zhao J., McDowell M. T., Lee H.-W., Zhao W., Cui Y. (2014). Nat. Nanotechnol..

[cit76] Clarke E. M., Wing J. M. (1996). ACM Comput. Surv..

[cit77] Tang H., Li W., Pan L., Tu K., Du F., Qiu T., Yang J., Cullen C. P., McEvoy N., Zhang C. (2019). Adv. Funct. Mater..

[cit78] Li Y., Arnold S., Husmann S., Presser V. (2023). J. Energy Storage.

[cit79] Yang K., Zhao F., Li J., Yang H., Wang Y., He Y. (2024). Adv. Funct. Mater..

[cit80] Zhao Q., Zhu Q., Miao J., Zhang P., Wan P., He L., Xu B. (2019). Small.

[cit81] Zhao M.-Q., Torelli M., Ren C. E., Ghidiu M., Ling Z., Anasori B., Barsoum M. W., Gogotsi Y. (2016). Nano Energy.

[cit82] Liu Y., Zhang P., Sun N., Anasori B., Zhu Q., Liu H., Gogotsi Y., Xu B. (2018). Adv. Mater..

[cit83] Tan B., Lu T., Luo W., Chao Z., Dong R., Fan J. (2021). Energ. Fuels.

[cit84] Li Z., Wang X. X., Zhang W., Yang S. (2020). Chem. Eng. J..

[cit85] Cao W. T., Ouyang H., Xin W., Chao S., Ma C., Li Z., Chen F., Ma M. G. (2020). Adv. Funct. Mater..

[cit86] Du Y., Wang X., Dai X., Lu W., Tang Y., Kong J. (2022). J. Mater. Sci. Technol..

[cit87] Cai X., Xiao Y., Zhang B., Yang Y., Wang J., Chen H., Shen G. (2023). Adv. Funct. Mater..

[cit88] Luo X., Zhu L., Wang Y. C., Li J., Nie J., Wang Z. L. (2021). Adv. Funct. Mater..

[cit89] Gao Y., Liu G., Bu T., Liu Y., Qi Y., Xie Y., Xu S., Deng W., Yang W., Zhang C. (2021). Nano Res..

[cit90] Bhatta T., Maharjan P., Cho H., Park C., Yoon S. H., Sharma S., Salauddin M., Rahman M. T., Rana S. S., Park J. Y. (2021). Nano Energy.

[cit91] Mirsepah A., Shooshtari L., Mohammadpour R., Esfandiar A., Irajizad A. (2024). Chem. Eng. J..

[cit92] Cao Y., Guo Y., Chen Z., Yang W., Li K., He X., Li J. (2022). Nano Energy.

[cit93] Anwer S., Umair Khan M., Mohammad B., Rezeq M., Cantwell W., Gan D., Zheng L. (2023). Chem. Eng. J..

[cit94] Gao Y., Liu G., Bu T., Liu Y., Qi Y., Xie Y., Xu S., Deng W., Yang W., Zhang C. (2021). Nano Res..

[cit95] Du Y., Wang X., Dai X., Lu W., Tang Y., Kong J. (2022). J. Mater. Sci. Technol..

[cit96] Murugesan R. A., Nagamuthu Raja K. C. (2023). Mater. Res. Bull..

[cit97] Huang H., Cui J., Liu G., Bi R., Zhang L. (2019). ACS Nano.

[cit98] Xu S., Wei G., Li J., Han W., Gogotsi Y. (2017). J. Mater. Chem. A.

[cit99] Ayman I., Rasheed A., Ajmal S., Rehman A., Ali A., Shakir I., Warsi M. F. (2020). Energ. Fuels.

[cit100] Nasrin K., Sudharshan V., Arunkumar M., Sathish M. (2022). ACS Appl. Mater. Interfaces.

[cit101] Cai Y. Z., Fang Y. S., Cao W. Q., He P., Cao M. S. (2021). J. Alloys Compd..

[cit102] Jiang H., Wang Z., Yang Q., Hanif M., Wang Z., Dong L., Dong M. (2018). Electrochim. Acta.

[cit103] Nasrin K., Sudharshan V., Subramani K., Karnan M., Sathish M. (2022). Small.

[cit104] Li L., Zhang N., Zhang M., Zhang X., Zhang Z. (2019). Dalton Trans..

[cit105] Liu Y., Zhou H., Zhou W., Meng S., Qi C., Liu Z., Kong T. (2021). Adv. Energy Mater..

[cit106] Yao Y., Zhang X., Tan L., Pan J., Zhan C., Liu W., Feng Y., Li H., Xiong L. (2024). ACS Appl. Nano Mater..

[cit107] Kumar S., Rehman M. A., Lee S., Kim M., Hong H., Park J. Y., Seo Y. (2021). Sci. Rep..

[cit108] Cheng W., Fu J., Hu H., Ho D. (2021). Adv. Sci..

[cit109] Zhao X., Peng L.-M., Tang C.-Y., Pu J.-H., Zha X.-J., Ke K., Bao R.-Y., Yang M.-B., Yang W. (2020). Mater. Horiz..

[cit110] Tan Y. Z., Wang H., Han L., Tanis-Kanbur M. B., Pranav M. V., Chew J. W. (2018). J. Membr. Sci..

[cit111] Wang H., Wu Y., Yuan X., Zeng G., Zhou J., Wang X., Chew J. W. (2018). Adv. Mater..

[cit112] Jamil F., Ali H. M., Janjua M. M. (2021). J. Energy Storage.

[cit113] Li K., Chang T.-H., Li Z., Yang H., Fu F., Li T., Ho J. S., Chen P.-Y. (2019). Adv. Energy Mater..

[cit114] Zhang P., Wang L., Du K., Wang S., Huang Z., Yuan L., Li Z., Wang H., Zheng L., Chai Z. (2020). J. Hazard. Mater..

[cit115] Jun B.-M., Park C. M., Heo J., Yoon Y. (2020). J. Environ. Manage..

[cit116] Kumar J. A., Prakash P., Krithiga T., Amarnath D. J., Premkumar J., Rajamohan N., Vasseghian Y., Saravanan P., Rajasimman M. (2022). Chemosphere.

[cit117] Rajan R., Rajasimman M., Natarajan R. (2010). Chem. Prod. Process Model..

[cit118] Thanh Hoai Ta Q., Park S., Noh J.-S. (2017). J. Colloid Interface Sci..

[cit119] Ibrahim Y., Meslam M., Eid K., Salah B., Abdullah A. M., Ozoemena K. I., Elzatahry A., Sharaf M. A., Sillanpää M. (2022). Sep. Purif. Technol..

[cit120] Lei Y., Cui Y., Huang Q., Dou J., Gan D., Deng F., Liu M., Li X., Zhang X., Wei Y. (2019). Ceram. Int..

[cit121] Jastrzębska A. M., Szuplewska A., Wojciechowski T., Chudy M., Ziemkowska W., Chlubny L., Rozmysłowska A., Olszyna A. (2017). J. Hazard. Mater..

[cit122] Rozmysłowska-Wojciechowska A., Szuplewska A., Wojciechowski T., Poźniak S., Mitrzak J., Chudy M., Ziemkowska W., Chlubny L., Olszyna A., Jastrzębska A. M. (2020). Mater. Sci. Eng. Carbon.

[cit123] Gu M., Dai Z., Yan X., Ma J., Niu Y., Lan W., Wang X., Xu Q. (2021). J. Appl. Toxicol..

[cit124] Wei Z., Peigen Z., Wubian T., Xia Q., Yamei Z., ZhengMing S. (2018). Mater. Chem. Phys..

[cit125] Li K., Zou G., Jiao T., Xing R., Zhang L., Zhou J., Zhang Q., Peng Q. (2018). Colloids Surf., A.

[cit126] Jun B.-M., Heo J., Taheri-Qazvini N., Park C. M., Yoon Y. (2020). Ceram. Int..

[cit127] Cai C., Wang R., Liu S., Yan X., Zhang L., Wang M., Tong Q., Jiao T. (2020). Colloids Surf., A.

[cit128] Sun B., Dong X., Li H., Shang Y., Zhang Y., Hu F., Gu S., Wu Y., Gao T., Zhou G. (2021). Sep. Purif. Technol..

[cit129] Tran N. M., Ta Q. T. H., Sreedhar A., Noh J.-S. (2021). Appl. Surf. Sci..

[cit130] Peng C., Wei P., Chen X., Zhang Y., Zhu F., Cao Y., Wang H., Yu H., Peng F. (2018). Ceram. Int..

[cit131] Mashtalir O., Cook K. M., Mochalin V. N., Crowe M., Barsoum M. W., Gogotsi Y. (2014). J. Mater. Chem. A.

[cit132] Zhang S., Liao S., Qi F., Liu R., Xiao T., Hu J., Li K., Wang R., Min Y. (2020). J. Hazard. Mater..

[cit133] Feng Y., Wang H., Xu J., Du X., Cheng X., Du Z., Wang H. (2021). J. Hazard. Mater..

[cit134] Li Y., Pan C., Kamdem P., Jin X.-J. (2020). Energy Fuels.

[cit135] Karthikeyan P., Ramkumar K., Pandi K., Fayyaz A., Meenakshi S., Park C. M. (2021). Ceram. Int..

[cit136] Zhang Z.-H., Xu J.-Y., Yang X.-L. (2021). Mater. Chem. Phys..

[cit137] Zhu Z., Xiang M., Shan L., He T., Zhang P. (2019). J. Solid State Chem..

[cit138] Luo S., Wang R., Yin J., Jiao T., Chen K., Zou G., Zhang L., Zhou J., Zhang L., Peng Q. (2019). ACS Omega.

